# Transduction of HEK293 Cells with BacMam Baculovirus Is an Efficient System for the Production of HIV-1 Virus-like Particles

**DOI:** 10.3390/v14030636

**Published:** 2022-03-18

**Authors:** Eduard Puente-Massaguer, Byron Cajamarca-Berrezueta, Aleix Volart, Irene González-Domínguez, Francesc Gòdia

**Affiliations:** Department of Chemical, Biological and Environmental Engineering, Universitat Autònoma de Barcelona, 08193 Cerdanyola del Vallès, Barcelona, Spain; byron.cajamarca@hotmail.com (B.C.-B.); aleix.volart@gmail.com (A.V.); irene.gonzalez@mssm.edu (I.G.-D.); francesc.godia@uab.cat (F.G.)

**Keywords:** BacMam baculovirus, HIV-1, virus-like particle, HEK293 cells, super-resolution fluorescence microscopy, endocytosis

## Abstract

Gag virus-like particles (VLPs) are promising vaccine candidates against infectious diseases. VLPs are generally produced using the insect cell/baculovirus expression vector system (BEVS), or in mammalian cells by plasmid DNA transient gene expression (TGE). However, VLPs produced with the insect cell/BEVS are difficult to purify and might not display the appropriate post-translational modifications, whereas plasmid DNA TGE approaches are expensive and have a limited scale-up capability. In this study, the production of Gag VLPs with the BacMam expression system in a suspension culture of HEK293 cells is addressed. The optimal conditions of multiplicity of infection (MOI), viable cell density (VCD) at infection, and butyric acid (BA) concentration that maximize cell transduction and VLP production are determined. In these conditions, a maximum cell transduction efficiency of 91.5 ± 1.1%, and a VLP titer of 2.8 ± 0.1 × 10^9^ VLPs/mL are achieved. Successful VLP generation in transduced HEK293 cells is validated using super-resolution fluorescence microscopy, with VLPs produced resembling immature HIV-1 virions and with an average size comprised in the 100–200 nm range. Additionally, evidence that BacMam transduction occurs via different pathways including dynamin-mediated endocytosis and macropinocytosis is provided. This work puts the basis for future studies aiming at scaling up the BacMam baculovirus system as an alternative strategy for VLP production.

## 1. Introduction

The rapid development of vaccines to tackle emerging infectious diseases has nowadays become more evident than ever with the current COVID-19 pandemic. Virus-like particles (VLPs) are an interesting vaccine platform since they are non-infective and non-replicative. Their ordered and repetitive structure mimicking the native virus likely contributes to an improved cellular immunity response in comparison to other vaccine platforms such as inactivated viruses and soluble recombinant proteins [1]. Proof of this is the already marketed VLP-based vaccines [2], as well as promising data for serious infectious diseases such as AIDS [3], Nipah virus [4], or COVID-19 pandemic [5], among significant others. In addition to the promising results of HIV-1 Gag VLPs in the development of an AIDS vaccine, the robust structure and ease of production of these nanoparticles in animal cells has opened the possibility to develop multivalent and chimeric vaccines [6,7]. The insect cell/baculovirus expression vector system (BEVS) has been widely exploited for the production of different VLP types [8] due to its high expression capacity, which is generally unrivalled, and its eased scale-up capability [9]. However, there are several limitations, such as the lytic nature of the baculovirus infection, releasing proteases that can degrade the product of interest. In some cases, the biochemical and immunological properties of the target product are compromised due to inaccurate post-translational modifications (i.e., sialylation) by insect cells. Additionally, the separation of baculovirus particles, which replicate efficiently in insect cells, from the co-produced enveloped VLPs might pose a serious hurdle to the purification process. Some efforts to produce VLPs in insect cells devoid of the BEVS have been recently reported, but although results seem promising, there is still a long way to go to achieve VLP titers in the range of the BEVS [10,11,12]. VLP production in human embryonic kidney 293 (HEK293) cells allows the circumventing of some of the aforementioned drawbacks of insect cell/BEVS, with recent studies reporting VLP titers in the same order of magnitude [13]. Nevertheless, plasmid DNA transient gene expression (TGE) is inefficient, uses expensive transfection reagents, and it is difficult to scale up [14]. Baculovirus are non-replicative in mammalian cells, but they are able to efficiently transduce these cells and express recombinant products when the genes of interest are under the control of mammalian promoters [15]. These genetically modified baculoviruses are commonly known as BacMam baculoviruses, and they can be used in multiple fields, such as gene therapy and drug discovery, besides vaccine development [16]. In this sense, this system provides an interesting alternative enabling efficient gene delivery and thorough post-translational modifications.

So far, there is little research and characterization on the production of complex VLPs with the BacMam expression system, with most of it using adherent cells or serum-containing media, thus hindering transference to higher volumes and bioprocess standardization. In this article, the production of HIV-1 Gag VLPs with the BacMam expression vector system in serum-free suspension HEK293 cells is assessed. The Gag protein is fused in frame with the enhanced green fluorescent protein (eGFP) to facilitate the quantification of cell transduction and VLP titers. The conditions providing the highest cell transduction and VLP titers are evaluated by means of Design of Experiments (DoE) and desirability functions. Different endocytic pathways for BacMam baculovirus entry in HEK293 cells are also studied in a drug inhibition assay. Finally, a complete characterization of the VLP production process and VLPs produced is performed, and the potential of this system as an alternative VLP production platform is discussed.

## 2. Materials and Methods

### 2.1. Cell Lines, Media, and Culture Conditions

HEK293 cells (HEK293SF-3F6) were cultured in 125 mL shake flasks (Corning, Corning, NY, USA) with 20 mL of supplemented Freestyle 293 medium (Thermo Fisher Scientific, Grand Island, NY, USA) containing 0.1% *v*/*v* Pluronic (Thermo Fisher Scientific), 19.8 mg/L of recombinant insulin (Novo Nordisk, Køge, Denmark), 1.6 mg/L of recombinant transferrin (MilliporeSigma, Burlington, MA, USA) and 0.9*X* mixture solution consisting of 0.9*X* of synthetic cholesterol, 0.9*X* of fatty acids, 1.8 µg/mL of tocopherol acetate, and 22.5 µg/mL of Tween 80 (MilliporeSigma) [17]. All the compounds used for FreeStyle 293 medium supplementation were animal-derived-component free. HEK293 cells were routinely maintained at viable cell concentrations in the range of 0.4–2 × 10^6^ cells/mL, 37 °C and shaken at 130 rpm in an 85% humified atmosphere of 5% CO_2_ in air.

Sf9 insect cells (cat. no. 71104, Merck, Darmstadt, Germany) were cultured in 125- or 1000-mL shake flasks with 20 or 250 mL of Sf900III serum-free medium (Thermo Fisher Scientific) at 27 °C and 130 rpm, respectively. Cells were routinely maintained at viable cell concentrations of 0.4–4 × 10^6^ cells/mL [10]. The viable cell concentration and viability of both cell lines was determined with a Nucleocounter NC-3000 (Chemometec, Allerød, Denmark) using acridine orange and 4′,6-diamidino-2-phenylindole according to manufacturer’s instructions.

### 2.2. BacMam Transfer Plasmid Construction

The BacMam baculovirus transfer vector pOET6 (Oxford Expression Technologies, Oxford, UK) encoding a Rev-independent HIV-1 Gag protein fused in frame to the enhanced GFP (cat. no. 11468, NIH AIDS Reagent Program) [18] was developed using the FlashBAC ULTRA expression system (Oxford Expression Technologies). The pOET6 transfer plasmid contains a CMV promoter that enables protein expression in mammalian cell lines. The pOET6-Gag-eGFP plasmid was constructed by enzymatic digestion of the pGag-eGFP [17] and pOET6 plasmids using the restriction enzymes *Not*I and *Kpn*I (Thermo Fisher Scientific).

### 2.3. Generation of BacMam Working Stock and Titration

Sf9 cells were transfected with the pOET6-Gag-eGFP transfer plasmid and the *Autographa californica* multicapsid nucleopolyhedrovirus (*Ac*MNPV) baculovirus genome (BaculoGold, BD Biosciences, Franklin Lakes, NJ, USA) upon complexation with Cellfectin II (Thermo Fisher Scientific). Supernatant containing BacMam viruses was harvested at 5 days post-transfection (P0) and maintained at 4 °C. The BacMam working stock (P3) was generated by three rounds of BacMam amplification at a multiplicity of infection (MOI) of 0.1 in Sf9 cells. The BacMam working stock was produced by infection of 1000 mL of Sf9 cell culture at 2–3 × 10^6^ cells/mL, and harvest by 3000× *g* centrifugation (Avanti J-20 XP Centrifuge, Beckman Coulter, Brea, CA, USA) for 10 min at 96 h post infection (hpi). The insect cell-derived supernatant containing BacMam baculoviruses was filtered using a Millex-HP 0.45 µm polyethersulfone syringe filter unit (MilliporeSigma), and medium was exchanged by precipitation with a polyethylene glycol (PEG) 10000 buffered solution. The PEG 10000 buffered solution consisted of 32% *w*/*v* of PEG 10000 (MilliporeSigma) in ultrapure water (MilliporeSigma), 400 mM NaCl (Thermo Fisher Scientific), 20 mM HEPES (MilliporeSigma), and pH adjusted to 7.4. The insect cell supernatant was mixed with the PEG 10000 buffered solution previously autoclaved at a ratio of 3:1. The mixture was kept at 4 °C overnight and subsequentially centrifuged at 4000 rpm for 30 min at 4 °C. Pellets containing BacMam baculoviruses were resuspended in fresh FreeStyle 293 supplemented medium and maintained at 4 °C. BacMam precipitation with the PEG 10000 buffered solution was performed twice to remove traces of the Sf900III insect cell medium.

The titration of the BacMam working stock was conducted by the plaque assay method. Briefly, 1 × 10^6^ Sf9 cell/well were plated in 6-well plates (Nunc, Thermo Fisher Scientific), and cells were left to attach at room temperature for 20–30 min. Then, the Sf900III medium was aspirated, and 1 mL/well of serial dilutions of BacMam baculovirus in Sf900III medium (10^−5^ to 10^−10^) was added. Plates were incubated at mild mixing conditions for 1 h to homogeneously distribute BacMam baculoviruses. Afterwards, the infection medium was aspirated, and 2 mL of overlay Sf900III medium containing 3% *w*/*v* low gelling temperature agarose (MilliporeSigma) was added per well to cover Sf9 cells. After agarose cooling, 2 mL of Sf900III culture medium supplemented with 10% *v*/*v* FBS were added per well. The plates were incubated at 27 °C for 5 days, and BacMam baculovirus plaques were measured by visual inspection after staining for 3 h with a 0.3% *w*/*v* Neutral Red solution prepared in Dulbecco’s phosphate-buffered saline (DPBS, Thermo Fisher Scientific).

### 2.4. Butyric Acid Toxicity Assays

The cytotoxicity effect of different concentrations of butyric acid on HEK293 cells was initially assessed using the Cell Titer AQ_ueous_ One Solution Cell Proliferation Assay (Promega, Madison, WI, USA). Briefly, 5 × 10^4^ HEK293 cells/well were seeded in 96-well plates, and 10 µL/well of different concentrations of butyric acid (0.5–24 mM) were added. After 48 h at 37 °C, 20 µL/well of Cell Titer 96 reagent was added and incubated at 37 °C for 1 h in the dark. A standard curve with known HEK293 cell concentrations ranging from 0 to 2 × 10^6^ cell/mL was also included the day of the analysis. The absorbance was measured in a Victor^3^ spectrophotometer (PerkinElmer, Waltham, MA, USA) at a wavelength of 490 nm.

Next, the effect of 3-, 5-, and 7-mM butyric acid concentrations on HEK293 cell viability was evaluated in shake flasks. HEK293 cells were seeded at a viable cell concentration of 2 × 10^6^ cell/mL in 20 mL of supplemented Freestyle 293 medium. An amount of 100 µL of butyric acid solution at each concentration was added. Viable cell concentration and cell viability were measured every 24 h.

### 2.5. VLP Production in HEK293 Cells

Gag-eGFP VLPs were produced by BacMam baculovirus transduction of HEK293 cells in 125 mL shake flasks. Different viable cell concentrations, multiplicities of infection, and butyric acid concentrations were tested. Butyric acid was added to HEK293 cells after 4 h post transduction (hpt) with the BacMam baculovirus. Samples were taken every 24 h to monitor viable cell concentration, cell viability, cell transduction and Gag-eGFP production. Samples were harvested by centrifugation at 1000× *g* for 5 min, and supernatants were kept at 4 °C until analysis.

### 2.6. D-Optimal Design

A computer-generated D-optimal design (MODDE Pro 12, Sartorius, Göttingen, Germany) was employed to analyze the effect of viable cell concentration, multiplicity of infection, and butyric acid concentration on two different responses, cell transduction efficiency (% of eGFP-positive cells) and VLP production (RFU, relative fluorescence units). The independent variables were screened at different levels (Table 1), which were linearly related for viable cell concentration and butyric acid concentration, and exponentially for multiplicity of infection, always in an equidistant manner. The results obtained for each response were fitted to a second-order polynomial equation by the least squares method as shown in Equation (1):

(1) $$Y=\beta_{0}+\sum \beta_{i}\cdot X_{i}+\sum \beta_{ij}\cdot X_{i}\cdot Xj+\sum \beta_{ii}\cdot X_{i}^{2}+\varepsilon$$

where *Y* is the response variable, *β_0_* is offset term, *β_i_* the linear coefficient, *β_ij_* the interaction coefficient, *β_ii_* the quadratic coefficient, *X_i_* and *X_j_* are the independent variables in coded values, and ε corresponds to the experimental error.

A multiple response optimization strategy based on desirability functions was employed to define a single optimal condition that considers both responses of the study. To this end, the functions corresponding to each response were transformed to a dimensionless scale *d* with values comprised between 0 and 1. The transformed functions were combined to maximize the overall desirability of the multiple response system [19].

### 2.7. Statistical Analysis

Statistical analysis of the different functions was conducted with the MODDE Pro 12 software. The coefficients R^2^ and R^2^_adj_ were used to assess the quality of the regression of the response functions. The overall significance of each function was determined by the analysis of variance (ANOVA) *F*-test, and the relevance of each individual coefficient was analyzed with the corresponding *t*-test. *p*-values of 0.05 or lower were considered as statistically significant.

### 2.8. Flow Cytometry

The percentage of Gag-eGFP transduced cells (eGFP-positive cells) was assessed in a BD FACS Canto II flow cytometer (BD Biosciences) equipped with a two-laser configuration (488 and 635 nm) [20].

### 2.9. Spectrofluorometry

The fluorescence of Gag-eGFP containing supernatant samples was assessed by spectrofluorometry. Supernatants were harvested by centrifugation at 1000× *g* for 5 min. Green fluorescence was measured in a Cary Eclipse Fluorescence Spectrophotometer at room temperature (Agilent Technologies, Santa Clara, CA, USA) set as follows: λ_ex_ = 488 nm (slit 5 nm), λ_em_ = 510 nm (slit 10 nm). RFU values were calculated by subtracting fluorescence unit values of non-transduced HEK293 supernatant samples (negative control). Fresh supplemented FreeStyle 293 medium, and a 0.1 mg/mL quinine sulphate solution were used as standards to normalize RFU values between experiments.

### 2.10. Nanoparticle Tracking Analysis

Gag-eGFP VLPs in the supernatant collected at 48 hpt were quantified using a NanoSight NS300 device (Malvern Panalytical, Malvern, UK) equipped with a 488 nm filter module for fluorescent nanoparticle detection. Samples were diluted in DPBS to achieve a final concentration of ~10^8^ particle/mL (20–60 particle/frame). Triplicate measurements of 60 s were analyzed with the NanoSight NTA 3.2 software (Malvern Panalytical).

### 2.11. Super-Resolution Confocal Microscopy

HEK293 cells transduced with BacMam baculovirus, and Gag-eGFP VLP supernatants harvested at 48 hpt were analyzed in a TCS SP8 confocal microscope (Leica Microsystems, Wetzlar, Germany). Briefly, cells were stained with 0.1% *v*/*v* CellMask and 0.1% *v*/*v* Hoechst 33342 (Thermo Fisher Scientific), which stained the plasma membrane in a deep red color and nucleic acids in cyan blue, respectively. After 10 min of incubation at room temperature, dye excess was removed by centrifugation at 300× *g* for 5 min and cell resuspension in fresh FreeStyle 293 medium. Samples were placed on glass bottom dishes (MatTek, Ashland, MA, USA) for visualization [21].

Gag-eGFP VLP supernatants were stained with 0.1% *v*/*v* CellMask. An amount of 50 µL of sample was placed on 76 × 26 mm microscope slides (Linea LAB, Barcelona, Spain) with 24 × 60 mm premier cover glass (Leica Microsystems) and incubated at room temperature for 30 min before imaging.

### 2.12. Electron Microscopy

The morphology and structure of VLPs from crude supernatants was assessed with two different electron microscopy techniques, including negative staining transmission electron microscopy (NS-TEM), and cryogenic transmission electron microscopy (cryo-TEM) as described previously [22]. For NS-TEM, 8 µL of sample were deposited on carbon-coated cooper grids (Micro to Nano, Wateringweg, The Netherlands) previously subjected to a glow discharge treatment (PELCO, Fresno, CA, USA) and incubated at room temperature for 1 min. Excess sample was carefully drained off the grid with the aid of filter paper. Then, 8 µL of 2% *w*/*v* uranyl acetate were added, and samples were incubated for 1 min at room temperature. Excess stained was drained off and grids were dried at room temperature until analysis. Sample visualization was performed in a JEM-1400 transmission electron microscope (JEOL, Akishima, Tokyo, Japan) equipped with an ES1000W Erlangshen charge-coupled device camera (model no. 785, Gatan, Pleasanton, CA, USA).

For cryo-TEM, 2 µL of sample were blotted on 400 mesh glow discharged Holey carbon grids (Micro to Nano). Samples were then plunged into liquid ethane at −180 °C using a Leica EM GP cryogenic workstation (Leica Microsystems) and visualized in a JEM-2011 electron microscope (JEOL) operating at 200 kV and equipped with a CCD multiscan camera (model no. 895, Gatan).

### 2.13. Inhibition of BacMam Baculovirus Uptake Pathways

The following compounds were used to selectively inhibit different endocytic pathways: 25 µM of chlorpromazine (clathrin-mediated endocytosis), 30 µM of nystatin (caveolin-mediated endocytosis), and 30 µM of *N*-ethyl-*N*-isopropyl amiloride (EIPA) (macropinocytosis), all of these compounds being prepared in ultrapure water (MilliporeSigma), and according to previous studies [23]. A fourth condition was also included to assess passive transport. In this case, HEK293 cells were cooled to 4 °C and maintained at this temperature for 4 h after BacMam baculovirus transduction. For the inhibition experiment, a medium replacement of HEK293 cells at 2 × 10^6^ cell/mL was initially performed in shaken 6-well plates, and then the specific inhibitors were incubated with cells for 2 h at 37 °C and 130 rpm. Afterwards, the BacMam baculovirus was added to the different conditions, and a second medium replacement was conducted at 4 hpt. Sampling to evaluate the impact on cell transduction efficiency and VLP production was performed at 48 hpt.

## 3. Results

### 3.1. Screening of Transduction Conditions

**Figure 1:**
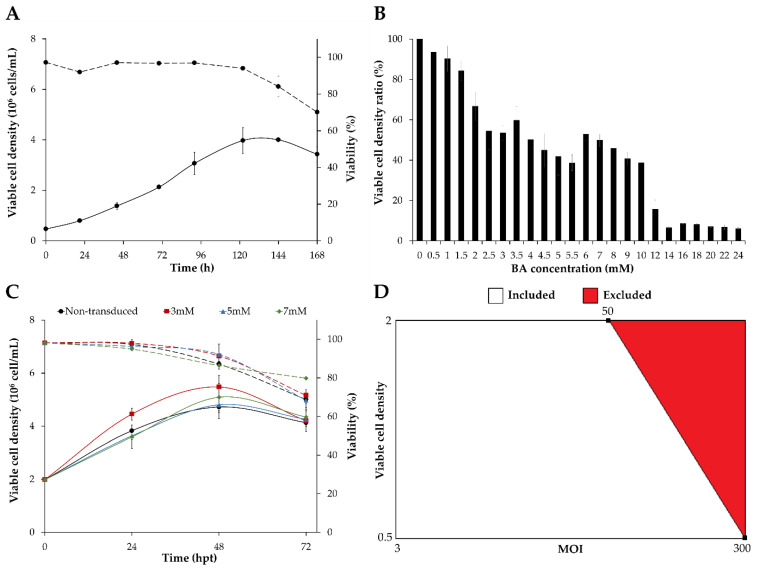
Definition of critical parameters for HEK293 cell transduction and D-optimal design. (**A**) Cell growth and viability profiles of HEK293 cells cultured in 20 mL of supplemented FreeStyle 293 medium. Cells were seeded at 0.5 × 10^6^ cell/mL in 125 mL shake flasks. (**B**) Cytotoxicity analysis of different butyric acid concentrations (0–24 mM) on HEK293 cells seeded at 5 × 10^4^ cell/well in 96-well plates. Data is expressed as the ratio of viable cell density at different BA concentrations compared with the no BA (0 mM) condition. (**C**) Addition of 3, 5 and 7 mM of butyric acid to HEK293 cells cultured in shake flasks with 20 mL of supplemented FreeStyle 293 medium and seeded at 2 × 10^6^ cell/mL. (**D**) Computer-generated D-optimal design. BA: butyric acid, hpt: hours post transduction, MOI: multiplicity of infection.

Different factors are known to impact on the efficiency of baculovirus transduction in adherent mammalian cell lines, including the multiplicity of infection (MOI), vesicular stomatitis virus G (VSV-G) protein, butyric acid (BA), incubation time, and incubation medium [24]. Variables such as the incubation time and the incubation medium are less relevant when using suspension-adapted cells due to impractical issues, but variables such as viable cell density (VCD) at transduction become more important to increase the final product titer. MOI, VCD, and BA concentration were selected as the critical parameters for HIV-1 Gag virus-like particle (VLP) production in suspension-adapted human embryonic kidney (HEK) 293 cells. Initially, the growth of HEK293 cells was assessed to determine the range of VCD at the exponential growth phase because lower transduction and production yields have been reported at late stages of cell growth [25]. Cells peaked at 4 × 10^6^ cell/mL with viabilities >90% at 5 days after seeding, and the VCD range at the exponential phase was defined as 0.5–2 × 10^6^ cell/mL (Figure 1A).

The effect of BA addition on HEK293 cell growth and viability was evaluated by supplementing cells with different concentrations (0.5–24 mM). Cell growth inhibition was observed in all the conditions tested, selecting the maximum BA concentration as the one resulting in a 50% reduction in cell growth (7 mM) compared to the non-supplemented condition (Figure 1B). BA concentrations in the range of 3–7 mM were additionally tested in shake flasks, resulting in no impact on cell growth and viability of HEK293 cells (Figure 1C). This BA concentration range was selected for next cell transduction experiments.

Preliminary experiments with BacMam baculovirus in Sf900III medium revealed widespread cell aggregation within few hours post transduction. After 2-time medium exchange of the BacMam baculovirus stock to FreeStyle 293 medium, cell clumping and a decline in cell viability after transduction at VCD of 2.0 × 10^6^ cells/mL and MOI > 100 could still be observed, although to a lesser extent (data not shown). Therefore, special attention was focused on selecting an appropriate design space for the Design of Experiments (DoE) phase taking into consideration this constraint. HEK293 cells were transduced with different MOI at two different VCD of 0.5 and 2 × 10^6^ cells/mL (Figure S1). Transductions of HEK293 cells at 2 × 10^6^ cells/mL were performed with MOI < 100 to overcome cell clumping. Cell viability decreased below 80% after 72 hpt especially in cell transduction conditions at 2 × 10^6^ cells/mL or MOI > 200, with remarkable cell clumping also observed when MOI > 300 and >60 were used at VCD of 0.5 and 2 × 10^6^ cells/mL, respectively. Best productions were obtained with a VCD of 2 × 10^6^ cells/mL and a MOI > 40, as well as those conditions with a VCD of 0.5 × 10^6^ cells/mL and a MOI > 100. Although higher VLP production titers were obtained at 72–96 hpt, a pronounced decline in cell viability and widespread cell clumping were detected, especially at higher MOI (Figure S1). Therefore, the harvest time was selected as 48 hpt to reduce the impact on product quality of the VLPs produced.

### 3.2. Optimization of HEK293 Cell Transduction by Design of Experiments (DoE)

**Figure 2:**
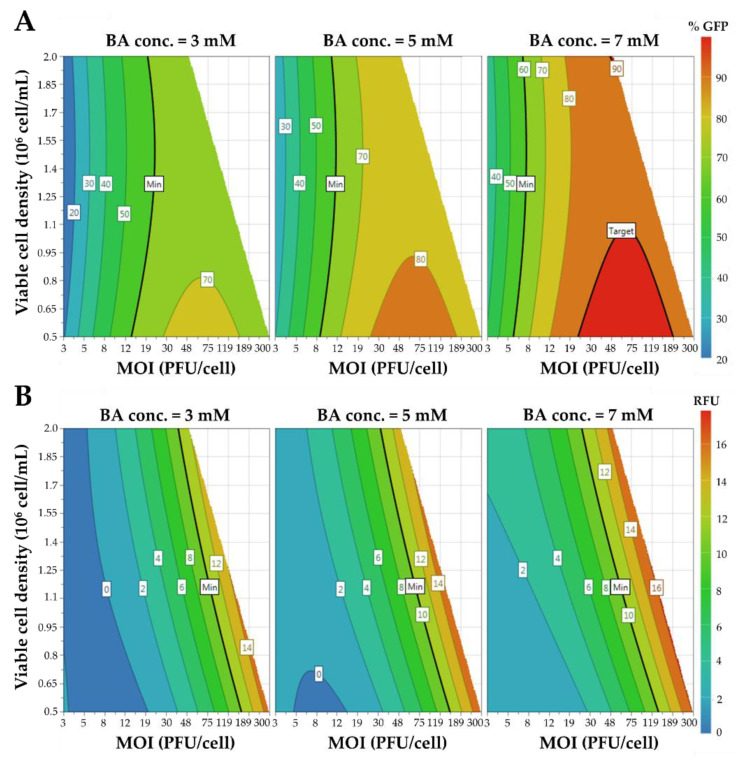
Contour plots of the different functions based on the D-optimal design. (**A**) Effect of VCD and MOI on HEK293 cell transduction efficiency at different BA concentrations. (**B**) Assessment of VCD and MOI on VLP production at different BA concentrations. BA: butyric acid, MOI: multiplicity of infection.

The synergistic effects of MOI, VCD, and BA concentration on HEK293 cell transduction efficiency and VLP production were studied using a DoE approach. A 3-variable computer-generated design was employed, taking especial consideration in minimizing the detrimental effect of combining high VCD and high MOI. To this end, an experimental constraint was set to exclude combinations of MOI > 50 with a VCD of 2 × 10^6^ cells/mL, and MOI > 300 when the VCD was 0.5 × 10^6^ cells/mL (Figure 1D). The computer-generated design consisted of 20 conditions (Table 1 and Table 2, Figure S2). The MOI and BA concentration were shown to be key for HEK293 cell transduction and VLP production, whereas the VCD was significantly relevant for VLP production only. The data were fitted to a second-order polynomial model (Equation (1)) by linear regression analysis for each of the two responses evaluated (Table 2), with $R^{2}$ and $R_{adj}^{2}$ coefficients of 0.979 and 0.972 for cell transduction efficiency, and 0.983 and 0.975 for VLP production, respectively. The statistical significance of both functions was confirmed by ANOVA analysis (Table 2). Contour plots were constructed for visual analysis of response trends to MOI, VCD and BA concentrations (Figure 2). MOI in the range of 20 to 200, and 7 mM BA concentration showed the highest cell transduction efficiencies (Figure 2A), while 7 mM BA concentration in combination with VCD and MOI near the experimental constraint provided the highest VLP titers (Figure 2B).

**Figure 3:**
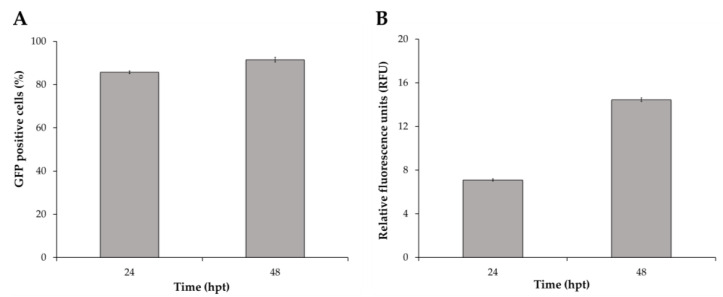
Validation experiment at the optimal conditions determined by the combination of DoE and desirability functions. (**A**) Monitoring of HEK293 cell transduction efficiency by flow cytometry, and (**B**) VLP production by spectrofluorometry up to 48 hpt. The mean and standard deviation of triplicate experiments are represented. hpt: hours post transduction.

Different MOI, VCD and BA concentration combinations resulted in the maximization of HEK293 cell transduction efficiency and VLP production. Thus, a global optimum condition that combined both functions was defined based on desirability functions [19], resulting in a MOI, VCD and BA concentration of 50 PFU/cell, 2 × 10^6^ cells/mL, and 7 mM, respectively. In these conditions, the predicted outcomes obtained by each modeled function were a cell transduction efficiency of 90.0 ± 5.6%, and a VLP production of 15.0 ± 1.5 RFU. A validation experiment was conducted at the optimal MOI, VCD and BA concentrations predicted by the models, resulting in a cell transduction efficiency of 91.5 ± 1.1%, and VLP production of 14.4 ± 0.2 RFU, in agreement with model predictions (Figure 3A,B). Analysis of VLP titer by nanoparticle tracking analysis (NTA), a technique that enables the direct measurement of nanoparticles in solution, yielded 2.8 ± 0.1 × 10^9^ VLPs/mL.

### 3.3. Characterization of VLP Production with the BacMam Expression System

**Figure 4:**
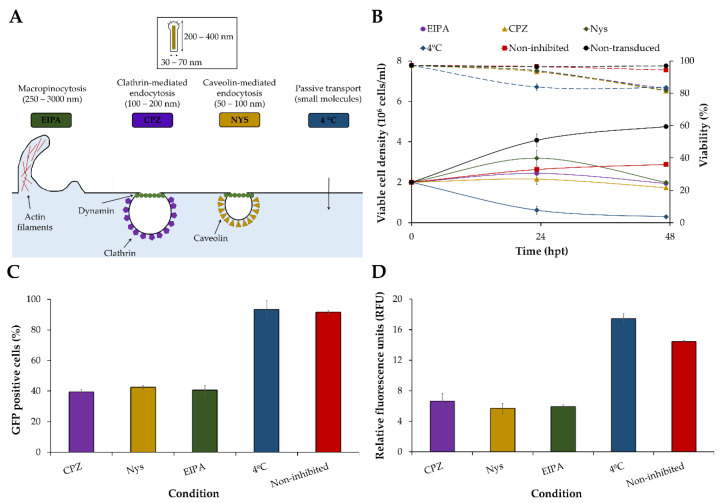
Inhibition of putative BacMam baculovirus endocytic pathways in a drug inhibition assay. (**A**) Schematic of different endocytic pathways. (**B**) Cell growth and viability profiles after drug inhibition of several endocytic pathways. (**C**) HEK293 cell transduction efficiency analyzed by flow cytometry. (**D**) VLP production assessed by spectrofluorometry. Chlorpromazine (CPZ, clathrin-mediated endocytosis), nystatin (Nys, caveolin-mediated endocytosis), *N*-ethyl-*N*-isopropyl amiloride (EIPA, macropinocytosis).

The uptake of BacMam baculoviruses by HEK293 cells was assessed in a drug inhibition assay. Baculoviruses have a width of 30–70 nm and a height of 200–400 nm, making different endocytic pathways the possible route for HEK293 cell transduction [26]. Macropinocytosis, clathrin-mediated endocytosis, and caveolin-mediated endocytosis were evaluated, with 5(*N*-ethyl-*N*-isopropyl) amiloride (EIPA), chlorpromazine (CPZ) and nystatin (NYS) used to inhibit each endocytic pathway, respectively (Figure 4A). Passive transport was also considered, with HEK293 cells maintained at 4 °C for 4 h after BacMam baculovirus transduction. A decline in cell viability could be observed at 48 hpt in all drug-inhibited conditions in comparison to non-transduced and non-inhibited controls (Figure 4B). Passive transport also showed a decline in cell growth after the treatment. A more than 2-fold reduction in HEK293 cell transduction and VLP production compared to the non-inhibited control was observed after inhibiting each endocytic pathway (Figure 4C,D). As expected, no impact on cell transduction and VLP production could be measured when passive transport was blocked.

**Figure 5:**
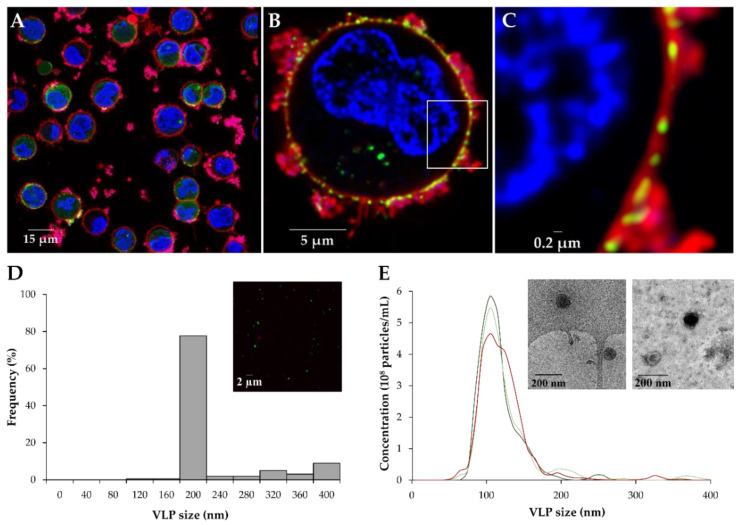
Assessment of VLP production in HEK293 cells and analysis of the VLPs produced at 48 hpt. (**A**) SRFM images of transduced HEK293 cells at 48 hpt. Cell nuclei were stained with Hoechst (blue), and the cytoplasmatic membrane was stained with CellMask^TM^ (red). (**B**) Deconvoluted image of a transduced HEK293 cell producing Gag-eGFP VLPs. (**C**) Zoom-in of the white section. (**D**) Analysis of VLP size by SRFM. Upper right: SRFM image of VLPs. (**E**) Analysis of VLP size using NTA. Upper right: cryo-TEM (left) and NS-TEM (right) images of VLPs.

The capacity of HEK293 cells to produce Gag-eGFP VLPs after BacMam baculovirus transduction was evaluated by super-resolution confocal microscopy (SRFM). Gag-eGFP production could be observed in most of HEK293 cells, corroborating efficient cell transduction with the BacMam expression system (Figure 5A). The generation of Gag-eGFP VLPs in the vicinity of the of plasma membrane could also be detected (green dots, Figure 5B,C). The size and morphology of Gag-eGFP VLPs was analyzed using different techniques that enable the characterization of nanoparticles in native conditions. SRFM was successfully applied for the quantification of BacMam-derived VLPs (Figure 5D, top right). The size of most of the Gag-eGFP VLPs produced was around 200 nm by SRFM, whereas the VLP size fell in the range of 80–150 nm when NTA was employed (Figure 5D,E). Analysis of the morphology of Gag-eGFP VLPs by NS-TEM and cryo-TEM also confirmed the spherical structure of these nanoparticles, resembling that of immature HIV-1 virions. Additionally, the presence of BacMam baculoviruses at 1.1 ± 0.1 × 10^7^ PFU/mL was detected by the plaque assay method.

## 4. Discussion

Since the discovery that baculovirus could transduce different mammalian cell lines without detectable baculovirus replication [27], different recombinant proteins have been produced with the BacMam expression system. However, the advantages of this system have not been fully exploited due to the limited studies available and the fact that most of these works are conducted in adherent cells [14,28], or serum-containing media [29,30], thus hindering bioprocess standardization and transference to higher volumes. In this work, the optimal transduction for HEK293 cells cultured in serum-free suspension conditions were determined, the VLP production process was characterized by means of novel analytical technologies, and different pathways were assessed for BacMam baculovirus endocytosis in HEK293 cells.

Preliminary screening experiments revealed that the addition of BacMam baculoviruses in the Sf900III medium at <10% of the final volume to be transduced induced high cell aggregation and severely compromised cell viability (data not shown). The differences in mammalian and insect cell culture media composition could be the reason explaining this detrimental effect on HEK293 cells. Therefore, a two-step medium replacement to FreeStyle 293 medium was conducted before HEK293 cell transduction to remove any traces of the Sf900III insect medium used to grow the BacMam baculovirus. Initial studies also showed the negative interaction of combining high MOI and high VCD at transduction, resulting in widespread cell aggregation. A computer-generated D-optimal design was used to minimize HEK293 cell aggregation and study the synergies between MOI, VCD, and BA acid addition on HEK293 cell transduction and VLP production. An MOI of 50, VCD at transduction of 2 × 10^6^ cells/mL, and BA concentration of 7 mM were determined as the best variable combination for HEK293 cell transduction and HIV-1 Gag VLP production on a combined DoE and desirability functions approach. In these conditions, a 91.5 ± 1.1% transduction of HEK293 cells and a VLP titer of 2.8 ± 0.1 × 10^9^ VLPs/mL was obtained. Attaining a high level of cell transduction is highly relevant for an easier bioprocess control and for maximizing production. Some studies report the production of different recombinant products, but there is no information about the transduction capacity of cultured cells, which makes bioprocess scale-up and optimization more difficult [31]. Moreover, some studies still use adherent cells or serum-containing media, and it is not always straightforward to transfer the optimal cell transduction conditions to suspension cell cultures [32]. Here, a cell transduction strategy attaining >90% transduced cells is reported for suspension HEK293 cells cultured in a serum-free medium.

Production of VLPs was more than 20-fold higher compared to the most recent study of BacMam baculovirus transduction of HEK293 cells [33]. VLP titers were also higher than those achieved by stable gene expression (SGE) [11,12] and transient gene expression (TGE) of insect cells [10,20], and higher or in the same range as SGE [34] and TGE [17] in HEK293 cells. VLP production with this system was lower in comparison to recent studies with the baculovirus expression vector system (BEVS) in insect cells [9,19]. However, a 20- to 40-fold reduction in BV particles was quantified with the BacMam expression system compared to the insect cell/BEVS, as well as a two- to three-fold enrichment in the ratio of VLPs to BV particles produced. It is well known that the production of enveloped VLPs with the insect cell/BEVS is accompanied by the co-production of contaminant BV particles that have similar physicochemical properties as VLPs, hindering their purification [35]. The BacMam expression system is an alternative strategy to significantly decrease the level of BV particles, while also easing the transference to higher production volumes as compared to plasmid DNA TGE methods. The release of proteases upon the lytic baculovirus infection of insect cells and subsequent degradation of target recombinant products is also abrogated [36]. Additionally, BacMam baculovirus-derived VLPs have been described as more immunogenic in comparison to VLPs produced by TGE in HEK293 cells [37]. Of course, there is the added effort of generating a high concentration BacMam baculovirus stock, but there is no requirement for expensive transfection reagents. A comparison of the BacMam expression system with other platforms used to produce HIV-1 Gag VLPs is provided in Table 3.

The successful generation of Gag-eGFP VLPs could be validated in the vicinity of the plasma membrane of HEK293 cells by SRFM. The results obtained in this work indicate that Gag-based VLPs might be a better scaffold for the production of chimeric or multivalent vaccines with the BacMam expression system, since M1-based VLPs were produced at lower yields [33]. The size and morphology of the VLPs produced were also analyzed using different techniques. The characteristic spherical particle surrounded by a bright corona could be measured by cryo-TEM and NS-TEM, resembling that of Gag VLPs produced in HEK293 cells by TGE and in the insect cell/BEVS [35]. VLP size was also in agreement with previous reports, with an average size of 110–120 nm by NTA, and around 200 nm when quantified by SRFM. Differences in VLP size by these two different techniques could be probably associated to the point spread function convolution effect of measuring small nanoparticles by SRFM [21].

Several endocytic pathways have been proposed for *Ac*MNPV infection and the transduction of insect and mammalian cells, respectively. These mainly involve clathrin- or other dynamin-mediated endocytosis [38], such as caveolin-dependent endocytosis [39], although some studies point in the direction of mammalian cell transduction via clathrin-independent pathways like macropinocytosis [40]. Here, we wanted to test whether one of these widely known endocytic pathways was predominant over the others for transducing suspension HEK293 cells. We observed that clathrin- and caveolin-mediated endocytosis, and macropinocytosis, but not passive transport, contributed to cell transduction once each of these pathways was specifically inhibited, thus indicating a compensation mechanism of endocytosis. Further studies should explore the simultaneous inhibition of multiple endocytic pathways to determine whether one of these pathways is more relevant than the others for HEK293 cell transduction. In any case, it is clear that BacMam baculovirus transduction is not dependent on a single pathway, which opens up the possibility of different supplementation strategies aiming at boosting VLP titers [41].

In summary, we describe and characterize the production of HIV-1 Gag VLPs with the BacMam expression system in suspension HEK293 cells, a system that combines the advantages of the insect cell/BEVS and plasmid DNA TGE. This work puts the basis for future scale-up studies with this system, as well as highlighting different molecular targets that could be employed to improve VLP titers.

## Figures and Tables

**Table 1:** Conditions of the independent variables studied in the D-optimal design.

Independent Variables	Coding Levels		
	−1	0	1
**MOI (PFU/cell)**	3	30	300
**Viable cell density (10^6^ cell/mL)**	0.5	1.25	2
**Butyric acid concentration (mM)**	3	5	7

**Table 2:** Matrix design, responses and ANOVA analysis for the D-optimal design at 48 hpt. Cell transduction efficiency (% of eGFP-positive cells) was measured by flow cytometry, and VLP production (RFU) by spectrofluorometry. Run 18, 19 and 20 are replicates to account for pure experimental error. Significant parameters (*p*-value < 0.05) are shown.

Exp. No.	MOI	VCD (10^6^ cell/mL)	Butyric Acid (mM)	Cell Transduction Efficiency (%)	VLP Production (RFU)
1	3	0.5	3	14.5	0.4
2	300	0.5	3	58.7	14.6
3	3	0.5	7	43.7	0.9
4	300	0.5	7	80.4	15.6
5	3	2	7	40.0	1.6
6	50	2	3	65.1	10.6
7	50	2	7	88.6	15.4
8	3	0.5	5.7	32.2	0.8
9	3	2	4.3	24.5	0.7
10	3	1	3	12.9	0.1
11	3	1.5	3	9.4	0.1
12	64.6	0.5	3	82.5	3.9
13	64.6	0.5	7	91.7	6.1
14	7.7	2	3	38.0	1.5
15	30	0.5	5	86.8	2.5
16	19.1	1.25	7	77.9	4.6
17	122.5	1.25	5	83.2	17.3
18	19.1	1.25	5	67.3	3.3
19	19.1	1.25	5	68.7	3.2
20	19.1	1.25	5	67.7	2.8
Cell transduction efficiency function—Parameters			Coefficient	Standard error	*p*-value
Constant			74.4	1.9	<0.01
MOI			20.9	1.8	<0.01
Butyric acid concentration			11.2	1.3	<0.01
MOI^2^			−30.9	2.7	<0.01
VLP production function—Parameters			Coefficient	Standard error	*p*-value
Constant			5.7	0.3	<0.01
MOI			11.5	0.5	<0.01
Viable cell density			4.0	0.3	<0.01
Butyric acid concentration			1.1	0.3	<0.01
MOI^2^			6.6	0.6	<0.01
MOI × Viable cell concentration			4.1	0.5	<0.01

**Table 3:** Comparison of different platforms employed to produce HIV-1 Gag VLPs. PTMs: post-translational modifications, BEVS: baculovirus expression vector system, BV: baculovirus, SGE: stable gene expression, TGE: transient gene expression. “+”: quantity of the property analyzed, “−”: no BV presence.

System	Cell Line	Titer	PTMs	Scalability	Cost	Time	BV Contamination
BacMam	HEK293	++	++++	++	++	++	+
SGE		++	++++	+++	++	++++	−
	Sf9	+	++	++++	+	+++	−
	High Five	+	++	++++	+	+++	−
TGE	HEK293	++	++++	+	++++	+	−
	Sf9	++	++	+	+++	+	−
	High Five	+	++	+	+++	+	−
BEVS		++++	++	+++	+	+	+++
	Sf9	++++	++	+++	+	+	++++

## Data Availability

Not applicable.

## Supplementary Materials

The following supporting information can be downloaded at: https://www.mdpi.com/article/10.3390/v14030636/s1, Figure S1: Preliminary HEK293 cell transduction experiment from 24 to 96 hpt. (A) Different MOI, VCD and butyric acid concentrations used. (B) Viable cell density. (C) Viability. (D) Transduction efficiency of HEK293 cells measured by flow cytometry. (E) Relative fluorescence units assessed by spectrofluorometry. NC 1: negative control consisting of non-transduced HEK293 seeded at 0.5 × 10^6^ cells/mL. NC 2: negative control consisting of non-transduced HEK293 seeded at 2 × 10^6^ cells/mL; Figure S2: Analysis of HEK293 cell transduction efficiency and VLP production in the different conditions of the DoE at 24 and 48 hpt. (A) Cell transduction was analyzed by flow cytometry, whereas (B) VLP production was assessed by spectrofluorometry.

## Abbreviations

BA: butyric acid; BEVS: baculovirus expression vector system; BV: baculovirus; cryo-TEM: cryogenic electron microscopy; eGFP: enhanced green fluorescent protein; FACS: fluorescence-activated cell sorting; HEK293: human embryonic kidney 293; HIV-1: human immunodeficiency virus serotype 1; hpi: hours post infection; hpt: hours post transduction; MOI: multiplicity of infection; NS-TEM: negative staining transmission electron microscopy; NTA: nanoparticle tracking analysis; RFU: relative fluorescence units; SGE: stable gene expression; SRFM: super-resolution fluorescence microscopy; TGE: transient gene expression; VCD: viable cell density; VLP: virus-like particle.
