# Supplementary material for: Transduction of HEK293 Cells with BacMam Baculovirus Is an Efficient System for the Production of HIV-1 Virus-like Particles
Source: Viruses. 2022 Mar 18;14(3):636. doi: 10.3390/v14030636 (PMC8954388; doi:10.3390/v14030636)
Supplement: Supplementary file 1 [file viruses-14-00636-s001.zip › viruses-1615122-supplementary.pdf]

Transduction of HEK293 cells with BacMam baculovirus is an efficient system for the production of HIV-1 virus-like particles

Eduard Puente-Massaguer [1,*], Byron Cajamarca-Berrezueta [1], Aleix Volart [1], Irene González-Domínguez [1], Francesc Gòdia [1]

[1] Department of Chemical, Biological and Environmental Engineering, Universitat Autònoma de Barcelona, Cerdanyola del Vallès, Barcelona, Spain

***Corresponding author:** Eduard Puente-Massaguer

E-mail: eduard.puente-massaguer@mssm.edu

Present address: Department of Microbiology, Icahn School of Medicine at Mount Sinai, 10029 New York, New York, USA

ORCID: 0000-0002-2816-7051

A

Exp. No.	MOI	VCD (10^6 cell/mL)	Butyric acid (mM)
1	10	0.5	3
2	20	0.5	3
3	30	0.5	3
4	50	0.5	3
5	70	0.5	3
6	100	0.5	3
7	200	0.5	3
8	300	0.5	3
9	400	0.5	3
10	2	2	3
11	5	2	3
12	10	2	3
13	20	2	3
14	30	2	3
15	40	2	3
16	50	2	3
17	70	2	3

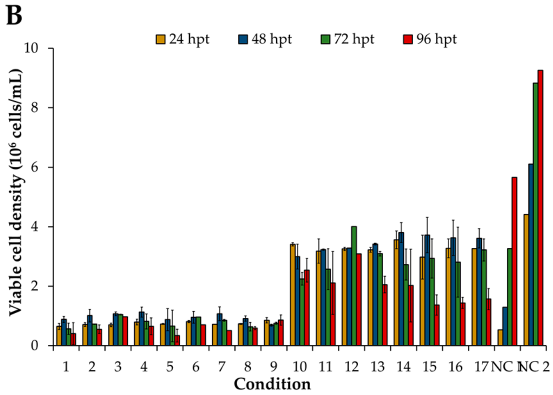

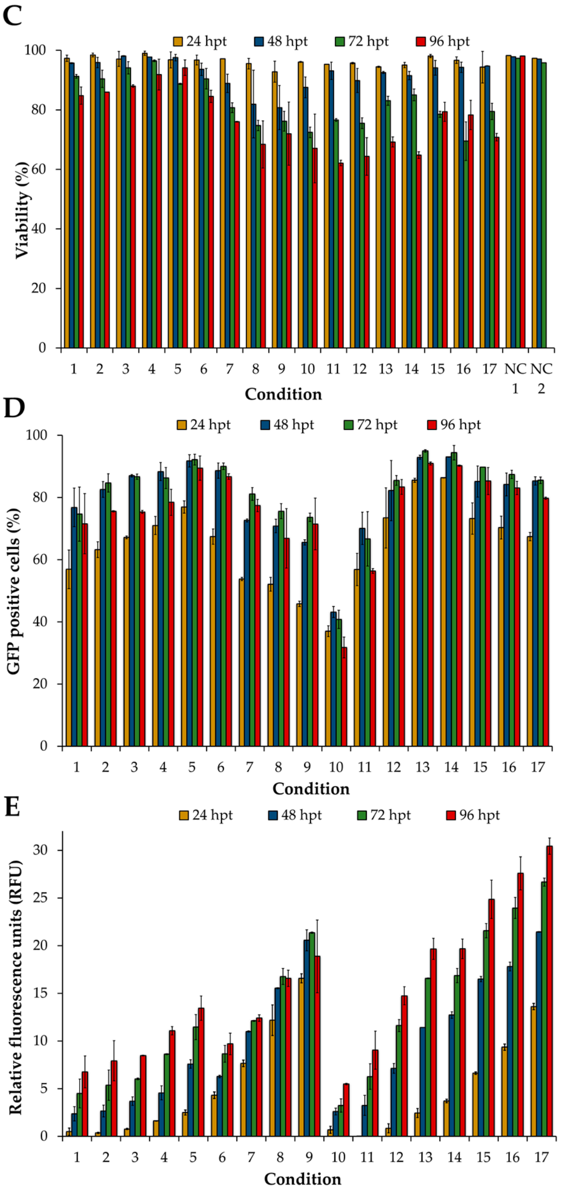

Figure S1. Preliminary HEK293 cell transduction experiment from 24 to 96 hpt. (A) Different MOI, VCD and butyric acid concentrations used. (B) Viable cell density. (C) Viability. (D) Transduction efficiency of HEK293 cells measured by flow cytometry. (E) Relative fluorescence units assessed by spectrofluorometry. NC 1: negative control consisting of non-transduced HEK293 seeded at 0.5 × 10^6 cells/mL. NC 2: negative control consisting of non-transduced HEK293 seeded at 2 × 10^6 cells/mL.

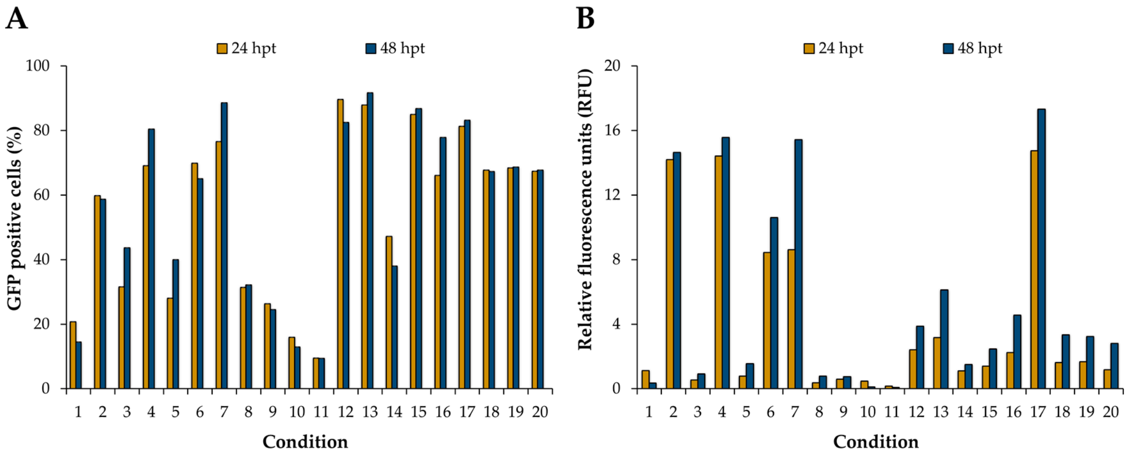

Figure S2. Analysis of HEK293 cell transduction efficiency and VLP production in the different conditions of the DoE at 24 and 48 hpt. (A) Cell transduction was analyzed by flow cytometry, whereas (B) VLP production was assessed by spectrofluorometry.
